# Human homolog of *Drosophila *Hairy and enhancer of split 1, Hes1, negatively regulates δ-catenin (*CTNND2*) expression in cooperation with E2F1 in prostate cancer

**DOI:** 10.1186/1476-4598-9-304

**Published:** 2010-11-24

**Authors:** Jian-Ping Lu, Jiao Zhang, Kwonseop Kim, Thomas C Case, Robert J Matusik, Yan-hua Chen, Michael Wolfe, Jongdee Nopparat, Qun Lu

**Affiliations:** 1 Department of Anatomy and Cell Biology, Brody School of Medicine, East Carolina University, Greenville, NC 27834 USA; 2 Leo Jenkins Cancer Center, Brody School of Medicine, East Carolina University, Greenville, NC 27834 USA; 3 College of Life Sciences, Zhejiang University, Hangzhou 310058 China; 4 College of Pharmacy, Chonnam National University, Gwangju, 500-757, Korea; 5 Department of Urological Surgery, Vanderbilt University Medical Center, Nashville, TN 37232 U.S.A; 6 Center for Neurologic Diseases, Brigham and Women's Hospital, Harvard Medical School, Boston, MA 02115 U.S.A

## Abstract

**Background:**

Neuronal synaptic junction protein δ-catenin (*CTNND2*) is often overexpressed in prostatic adenocarcinomas but the mechanisms of its activation are unknown. To address this question, we studied the hypothesis that Hes1, human homolog of *Drosophila *Hairy and enhancer of split (Hes) 1, is a transcriptional repressor of δ-catenin expression and plays an important role in molecular carcinogenesis.

**Results:**

We identified that, using a *δ-catenin *promoter reporter assay, Hes1, but not its inactive mutant, significantly repressed the upregulation of δ-catenin-luciferase activities induced by E2F1. Hes1 binds directly to the E-boxes on *δ-catenin *promoter and can reduce the expression of δ-catenin in prostate cancer cells. In prostate cancer CWR22-Rv1 and PC3 cell lines, which showed distinct δ-catenin overexpression, E2F1 and Hes1 expression pattern was altered. The suppression of Hes1 expression, either by γ-secretase inhibitors or by siRNA against Hes1, increased δ-catenin expression. γ-Secretase inhibition delayed S/G2-phase transition during cell cycle progression and induced cell shape changes to extend cellular processes in prostate cancer cells. In neuroendocrine prostate cancer mouse model derived allograft NE-10 tumors, δ-catenin showed an increased expression while Hes1 expression was diminished. Furthermore, *E2F1 *transcription was very high in subgroup of NE-10 tumors in which *Hes1 *still displayed residual expression, while its expression was only moderately increased in NE-10 tumors where Hes1 expression was completely suppressed.

**Conclusion:**

These studies support coordinated regulation of δ-catenin expression by both the activating transcription factor E2F1 and repressive transcription factor Hes1 in prostate cancer progression.

## Background

Deregulation of gene expression is one of the most prominent features of cancer. Upregulated or downregulated genes interfere with signaling pathways leading to altered cell functions. Thus, the elucidation of different mechanisms responsible for changes in gene expression is essential for the understanding of tumorigenesis.

δ-Catenin (*CTNND2*) or NPRAP (neural plakophilin-related arm-repeat protein) was first identified with its primary expression in neural and neuroendocrine tissues [1]. Many studies showed that δ-catenin expression is tightly controlled, and the alteration of its expression level is associated with a number of human diseases. The hemizygous deletion of *δ-catenin *gene is associated with the severe mental retardation phenotype of Cri-du-Chat syndrome [2]. On the other hand, increased δ-catenin expression modifies adhesion molecules, reshapes cellular morphology, and promotes cell migration [3,4]. Most strikingly, δ-catenin was found to be overexpressed in several cancers of peripheral tissues, including prostate, esophagus, and breast tumors [5], and upregulated in the majority of prostatic adenocarcinomas [6]. Overexpressed δ-catenin can be detected in urine and is accumulated significantly in prostate cancer patients [7]. Increased expression of δ-catenin disrupts cell-cell junctions [3,6] and promotes human prostate cancer cell growth and tumor progression, altering cell cycle and survival gene profiles [8].

Increased expression of δ-catenin in carcinogenesis is modulated by multiple mechanisms, including gene amplification, transcriptional activation, and mutation in its non-coding region [9]. It was reported that E2F1 positively regulates the expression of δ-catenin in human prostate cancer cells [10], and the expression of both genes is altered in prostate cancer [6,11]. On the other hand, Hes1, human homolog of *Drosophila *Hairy and enhancer of split 1, and a basic helix-loop-helix (bHLH) transcriptional repressor, shows potential binding sites on human *δ-catenin *promoter region near that of E2F1 [10]. Hes1 is a target gene of Notch1 activation, which is believed to be critical for the development of prostate cancer [12,13]. In transgenic mouse models of prostate cancer, NE-10 prostate tumor from subcutaneous transplantation of 12T-10 tumor and CR2-TAg prostate, Notch-Hes1 signaling is down-regulated and may be responsible for the promotion of the neuroendocrine differentiation of prostate cancer cells [14,15].

δ-Catenin is upregulated in human prostate cancer [6], and Hes1 expression is altered in tumorigenesis [16,17]. However, the ability of Hes1 to inhibit the expression of δ-catenin in prostate cancer cells and the cooperation between Hes1 and other transcription factors for modulating δ-catenin expression in prostate development and tumorigenesis are still unclear. In this study, we demonstrated that Hes1 is a transcriptional repressor for *δ-catenin *and regulates δ-catenin expression in human prostate cancer cells and mouse models of prostate tumors by coordinating with transcription activator E2F1.

## Results

### Hes1 inhibits the upregulation of δ-catenin-luciferase activities induced by E2F1 in prostate cancer cells

**Figure 1:**
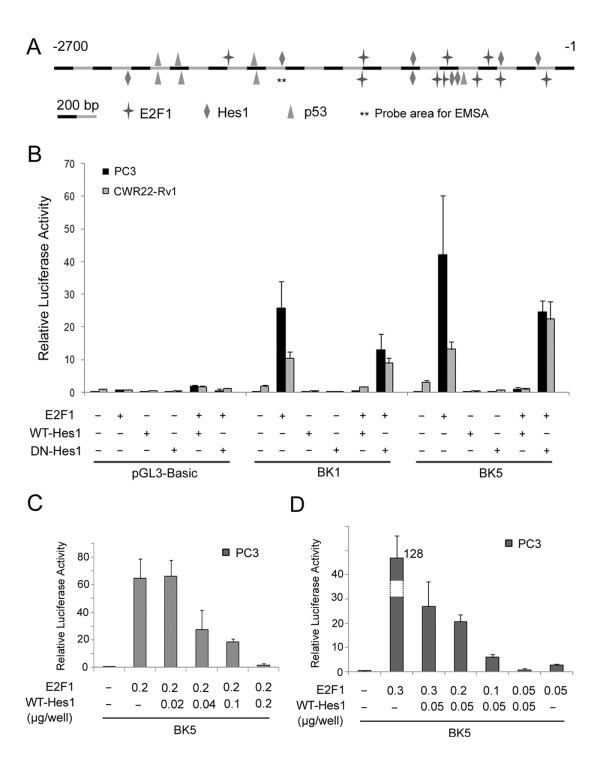
**Hes1 inhibits δ-catenin transcription in prostate cancer cells**. (A) A schematic graph showing predicted binding regions of Hes1, E2F1, and p53 on δ-catenin promoter. Numbers on top indicate the sequence location in base pairs from the transcription start site. (B) Hes1 is a negative regulator of *δ-catenin *transcription. *E2F1, WT-Hes1 *and *DN-Hes1 *expression vectors together with two human *δ-catenin-luciferase *reporter vectors (BK1 and BK5) and a control vector (*pGL3-Basic*), were co-transfected into PC3 and CWR22-Rv1 cells (0.2 μg/well for each vector in 12-well plates) as indicated. Hes1 blocked δ-catenin-luciferase activity completely, which was induced by E2F1. DN-Hes1 did not block E2F1 induced δ-catenin-luciferase activity, regardless of cell types and reporter vectors used. (C and D) Hes1 inhibited δ-catenin-luciferase activity (BK5 reporter vector) in a dose-dependent manner. PC3 cells were co-transfected with *pcDNA-flag-WT-Hes1 *and E2F1 vectors. (C) E2F1 plasmid was used at 0.2 μg/well and *pcDNA-flag-WT-Hes1 *plasmid was used for co-transfection at 0.02 μg, 0.04 μg, 0.1 μg and 0.2 μg per well separately in 12-well plates. (D) *pcDNA-flag-WT-Hes1 *plasmid was used at 0.05 μg/well and *E2F1 *plasmid was used for co-transfection at 0.3 μg, 0.2 μg, 0.1 μg and 0.05 μg per well separately in 12-well plates. The data was representative of three independent experiments.

Human *δ-catenin *promoter region contains multiple potential binding sites for positive or negative regulators revealed by Genomatix program http://www.genomatix.de/[10]. Among them, E2F1 has been identified as a positive regulator of δ-catenin expression in prostate cancer cells. On the other hand, Hes1 was predicted to have several binding sites near the binding sites of E2F1 on *δ-catenin *promoter (Figure 1A). BK1 and BK5 were two δ-catenin-luciferase reporter vectors, containing 2664 bp and 744 bp of *δ-catenin *promoter, respectively. When co-transfected with *E2F1 *expression vector, δ-catenin-luciferase activities were dramatically increased in prostate cancer cell lines [10]. To test the hypothesis that Hes1 is a negative regulator of δ-catenin expression, we co-transfected *Hes1 *expression vectors (*pcDNA-flag-WT-Hes1 *or *pcDNA-flag-DN-Hes1*) and *E2F1*, together with *δ-catenin-luciferase *reporter vectors BK1 or BK5, into CWR22-Rv1 or PC3 human prostate carcinoma cell lines. As shown in Figure 1B, E2F1 induced dramatic increases in δ-catenin-luciferase activities in both cell lines with either BK1 or BK5 co-transfection. After co-transfection with *WT-Hes1 *expression vector, E2F1-induced δ-catenin-luciferase activities were completely blocked in both cell lines (Figure 1B). We then applied a dominant negative mutant Hes1 (*DN-Hes1*), where amino acids E43, K44, and R47 in the basic region were each mutated to A. Literature indicated that DN-Hes1 cannot bind to DNA but can still dimerize with the endogenous WT-Hes1 to form a non-DNA-binding heterodimer complex [18]. DN-Hes1 led to a moderate reduction in E2F1-induced δ-catenin-luciferase expression in PC3 (Figure 1B) that did not reach statistical significance. No change was found in CWR22-Rv1 cells (Figure 1B). The negative effects of *Hes1 *on *E2F1*-induced δ-catenin-luciferase activities were dose-dependent (Figure 1C). Furthermore, when Hes1 expression was kept constant, increasing E2F1 expression also showed dose-dependent changes in δ-catenin-luciferase activities (Figure 1D). These results demonstrated that Hes1 can functionally interact with E2F1 in controlling δ-catenin expression in prostate cancer cells.

### Hes1 interacts with δ-catenin promoter

**Figure 2:**
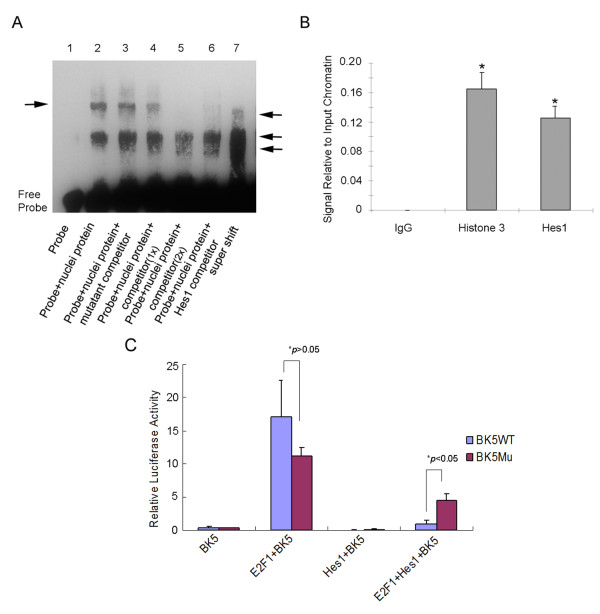
**Hes1 interacts with δ-catenin promoter**. (A) EMSA to show direct binding of Hes1 to *δ-catenin *promoter. Lane 1, control (labeled *δ-catenin *promoter probe spanning the HLH motif, marked as ** in the Figure 1A); lane 2, labeled probe with WT-Hes1 overexpressed nuclear extracts, forming DNA-protein complexes (indicated by arrow); lane 3, unlabeled probe with E-box mutated, showing DNA-protein complexes; lane 4, unlabeled competitor + nuclear extracts + labeled probe, low dose unlabeled probe reduced the binding of protein-DNA complexes; lane 5, unlabeled competitor + nuclear extracts + labeled probe, high dose unlabeled probe inhibited the binding of protein-DNA complexes; lane 6-7, gel shift immune-assays were performed with the same labeled probe using an anti-Hes1 antibody. Incubating Hes1-overexpressed nuclear extracts with anti-Hes1 antibody before the addition of probe DNA inhibited protein-DNA complexes (lane 6). Incubating Hes1-overexpressed nuclear extracts with anti-Hes1 antibody after the addition of probe DNA disrupted the slowest moving protein-DNA complexes (lane 7, arrow). However, under this experimental condition, supershifts did not occur but the partially disrupted protein-DNA complex resulting fast moving band accumulation can be detected (lane 7, double arrows). (B) Anti-Hes1 chromosome immunoprecipitation (ChIP) of the *δ-catenin *promoter. Real-time qRT-PCR showed the signal relative to input chromatin. Compared with the negative control experiment using IgG, anti-Hes1 greatly recruited the *δ-catenin *promoter DNA. Anti-Histone H3 ChIP was performed as a positive control. *: *p *< 0.05. (C) Hes1 binding to E-boxes on *δ-catenin *promoter is important to negatively regulate E2F1 induced *δ-catenin-luciferase *reporter activity. Note: E2F1 expression elicited a strong *δ-catenin-luciferase *reporter activity in PC3 cells, whatever BK5 sequence was mutated (*p > 0.05). But when WT-Hes1 co-transfection with E2F1, it significantly suppressed E2F1 induced *δ-catenin-luciferase *reporter activity when wild type *δ-catenin *promoter sequence BK5 was employed compared to BK5 sequence mutated vector (*p <0.05).

To explore whether Hes1 suppresses δ-catenin expression by directly binding to *δ-catenin *promoter, we performed electrophoretic mobility shift assay (EMSA). *δ-Catenin *promoter contains several E-boxes and N-boxes (Figure 1A). We applied biotin to label and prepare a 60 bp dsDNA oligonucleotide probe (marked as ** in Figure 1A) spanning one E-box. Nuclear extracts were prepared from PC3 or CWR22-Rv1 cells that were transiently transfected with Hes1 for 24 hours. EMSA demonstrated a clearly shifted protein-DNA complex after incubating the labeled probes with nuclear extracts from cells overexpressing WT-Hes1 (Figure 2A, lane 2, and arrow). This shift was also observed when the E-box was mutated in the unlabeled probes and incubated with nuclear extracts of cells overexpressing WT-Hes1 (Figure 2A, lane 3, arrow). Competition experiments showed that the unlabeled *δ-catenin *promoter sequences applied at low dose reduced Hes1 protein*-δ-catenin *promoter complexes (Figure 2A, lane 4, arrow), whereas that of high dose completely inhibited the Hes1 protein*-δ-catenin *promoter complexes (Figure 2A, lane 5, arrow). To determine whether Hes1 was responsible for the formation of the shifted protein-DNA complexes, we added anti-Hes1 antibody into the nuclear extracts followed by the gel-shift immunoassays. After incubating Hes1-overexpressed nuclear extracts with anti-Hes1 antibody before the addition of DNA probe, protein-DNA complexes related to Hes1*-δ-catenin *promoter disappeared (Figure 2A, lane 6, arrow). In other experiments, incubation of nuclear extracts with normal IgG or antibodies against non-relevant proteins did not reduce shifted bands (data not shown). When anti-Hes1 was added after the probe was incubated with the nuclear extracts, the slowest moving protein-DNA complexes were disrupted. However, under this experimental condition, supershifts did not occur but the partially disrupted protein-DNA complex can be detected (Figure 2A, lane 7, double arrows).

We have also performed the ChIP assay to further examine the interaction of endogenous Hes1 protein with *δ-catenin *promoter. The signal relative to input chromatin revealed by real-time PCR showed that anti-Hes1 recruited the *δ-catenin *promoter DNA to the similar levels to the positive control using anti-Histone 3 (Figure 2B). These combined results including EMSA and CHIP demonstrated that endogenous as well as ectopically transfected Hes1 protein is capable of direct binding to the *δ-catenin *promoter.

Additionally, to determine whether Hes1 binding domain on *δ-catenin *promoter is functional, we examined the ability of Hes1 protein to inhibit *δ-catenin-luciferase *reporter activity when selective, putative Hes1 binding E-boxes on *δ-catenin *promoter were mutated (Figure 2C). As shown in Figure 2C, E2F1 expression elicited a strong *δ-catenin-luciferase *reporter activity in PC3 cells, whether the BK5 sequence was mutated or not. WT-Hes1 co-transfection with E2F1 significantly suppressed E2F1 induced *δ-catenin-luciferase *reporter activity when wild type *δ-catenin *promoter sequence BK5 was employed. However, the ability of WT-Hes1 to suppress E2F-1 induced *δ-catenin-luciferase *reporter activity was reduced when BK5 sequence was mutated (*p *< 0.05) (Figure 2C). Therefore, Hes1 was able to bind to *δ-catenin *promoter and repress its activity through E-boxes, although other potential Hes1 binding sites may play additional suppressive roles on *δ-catenin *promoter.

### Prostate cancer cell lines display altered expression of δ-catenin and its potential transcription regulators

**Figure 3:**
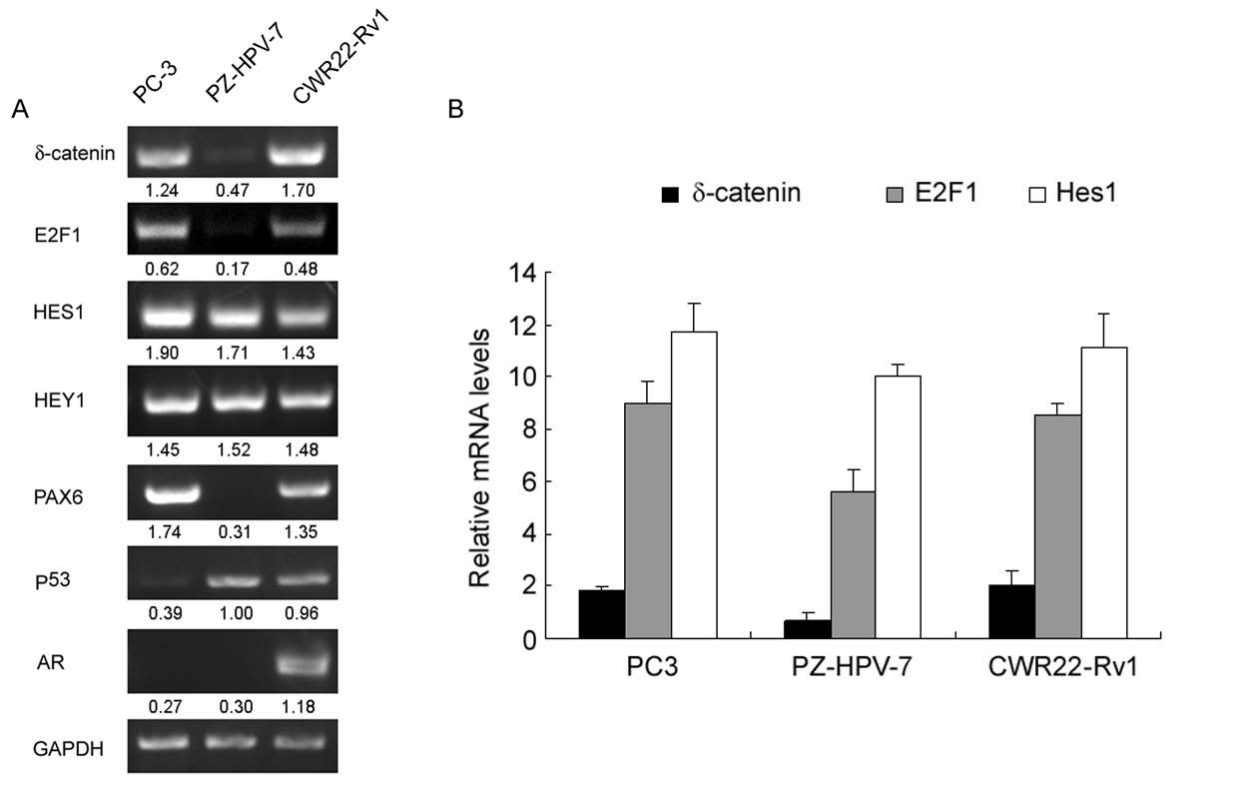
**RT-PCR and real-time qRT-PCR analyses of mRNA expression of δ-catenin and its potential transcriptional regulators**. (A) RT-PCR. Total RNAs were prepared from human prostate cancer cell lines (PC3 and CWR22-Rv1) and a non-cancer prostate cell line (PZ-HPV-7). RT-PCR analyses were performed to determine the mRNA expression of *Hes1, Hey1, E2F1, Pax6, p53, AR*, and *δ-catenin *genes. *GAPDH *was used as control. Numbers beneath each lane are the semi-quantification of RT-PCR data normalizing to *GAPDH*. (*p *< 0.05) (B) Real-time qRT-PCR analyses of relative mRNA levels of *E2F1, Hes1*, and *δ-catenin *in comparison to *GAPDH*. While the trends were clear that the transcript levels of *E2F1 *and *Hes1 *were higher in PC3 cells than that in CWR22-Rv1 cells, they were not statistically significant. Three independent experiments were performed.

Our previous studies demonstrated δ-catenin upregulation in prostate cancer by both RT-PCR and immunohistochemistry [6]. Overexpression of δ-catenin in prostatic adenocarcinomas could be due to increased activities of its positive transcriptional regulators and/or decreased activities of its negative transcriptional regulators. To further exploit the potential transcriptional regulators of δ-catenin in prostate cancer cells, we applied RT-PCR to compare the expression patterns of potential transcription factors in CWR22-Rv1 and PC3, two prostate cancer cell lines that show distinctly higher δ-catenin expression compared to the non-cancer prostate epithelial cell line PZ-HPV-7 (Figure 3A, δ-catenin). Coincident with δ-catenin expression, *E2F1 *expression correlated very well with CWR22-Rv1 and PC3 while it was barely detected in PZ-HPV-7 (Figure 3A, E2F1). *Hes1 *expression was clearly detected in all of these cell lines. *Hey1*, another *Hes *related family of bHLH type transcriptional repressors, had a similar transcript distribution pattern in these cells compared with that of *Hes1 *(Figure 3A, Hes1 and Hey1). Real-time qRT-PCR confirmed the RT-PCR results (Figure 3B). Pax6, another known positive transcription factor for *δ-catenin *[10], showed similar changes to *E2F1 *in CWR22-Rv1 cells and PC3 cells compared with PZ-HPV-7 cells (Figure 3, Pax6). The expression of p53 and androgen receptor (AR) did not show clear correlation with *δ-catenin *expression. These data indicate that E2F1 and Hes1 may influence *δ-catenin *transcript level in prostate cancer PC3 or CWR22-Rv1 cells.

### Overexpression of Hes1 can reduce the expression of δ-catenin in prostate cancer cells

**Figure 4:**
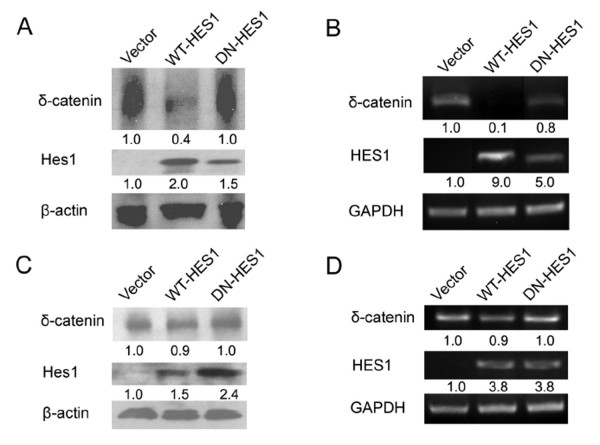
**Overexpression of Hes1 suppressed the δ-catenin expression**. (A) Western blots showing the expression of δ-catenin, Hes1, and actin in total lysates of CWR22-Rv1 cells transfected with *WT-Hes1, DN-Hes1 *or *pcDNA3 *(vector as control), respectively. (B) RT-PCR analysis of the expression of *δ-catenin*, *Hes1*, and *GAPDH *genes in CWR22-Rv1 cells transfected with *WT-Hes1, DN-Hes1 *or *pcDNA3*, respectively. (C) Western blots showing the expression of δ-catenin, Hes1, and actin in total lysates of PC3 cells transfected with *WT-Hes1, DN-Hes1 *or *pcDNA3*, respectively. (D) RT-PCR analysis of *δ-catenin*, *Hes1*, and *GAPDH *gene expression in PC3 cells transfected with *WT-Hes1, DN-Hes1 *or *pcDNA3*, respectively. Note: Actin or GAPDH as either Western blot or RT-PCR control. The numbers beneath each gel lane reflects the relative intensity compared to vector control and normalized against actin or GAPDH (*p *< 0.05). Both the Western blot and RT-PCR experiments were repeated at least three times.

We then examined whether ectopic overexpression of *Hes1 *can act as a negative regulator of δ-catenin expression in prostate cancer cells. When *pcDNA-flag-WT-Hes1 *was transfected into CWR22-Rv1 cells, both δ-catenin mRNA and protein levels were reduced dramatically compared to control and *DN-Hes1 *(Figure 4A and 4B). The same trends can be observed in PC3 cells, however the reduction of δ-catenin mRNA and protein was more moderate (only 10%, *p *< 0.05) (Figure 4C and 4D).

**Figure 5:**
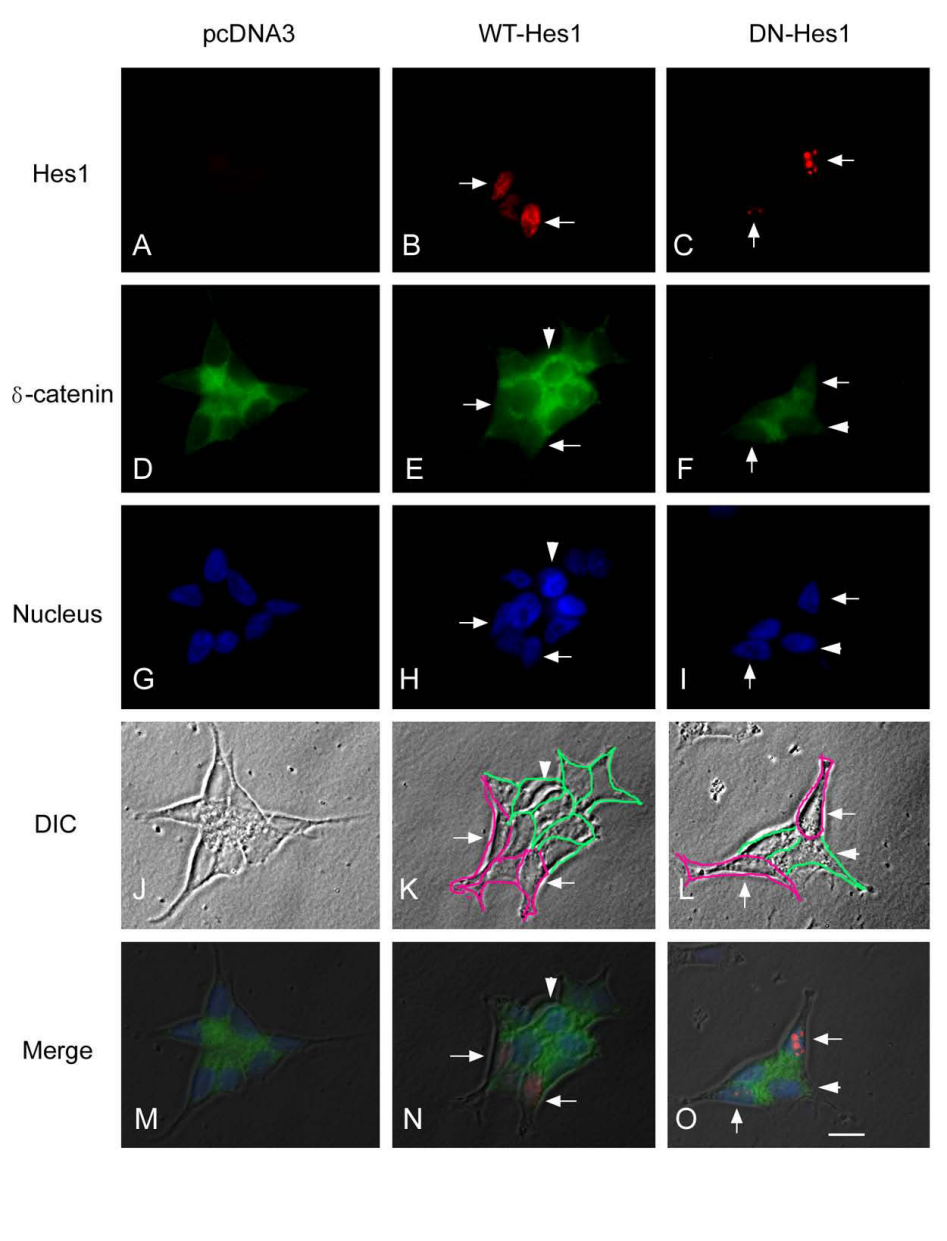
**Immunofluorescence light microscopy of CWR22-Rv1 cells transfected with WT-Hes1, DN-Hes1 or pcDNA3, respectively**. Red shows Hes1 protein stained by anti-flag-M5 antibody. Green shows δ-catenin stained by R1152 antibody. Blue shows nuclei stained with Hoechst 33258. To better reveal the cell-cell boundary in otherwise clustered CWR22-Rv1 cells, purple lines were drawn to indicate the cells transfected while the green lines marked the cells untransfected in K and L. Arrows (B, E, H, K and N): cells transfected with *WT-Hes1*; Arrows (C, F, I, L and O): *DN-Hes1*. Arrowheads: cells transfected with *pcDNA3*. Bar: 10 μm.

**Figure 6:**
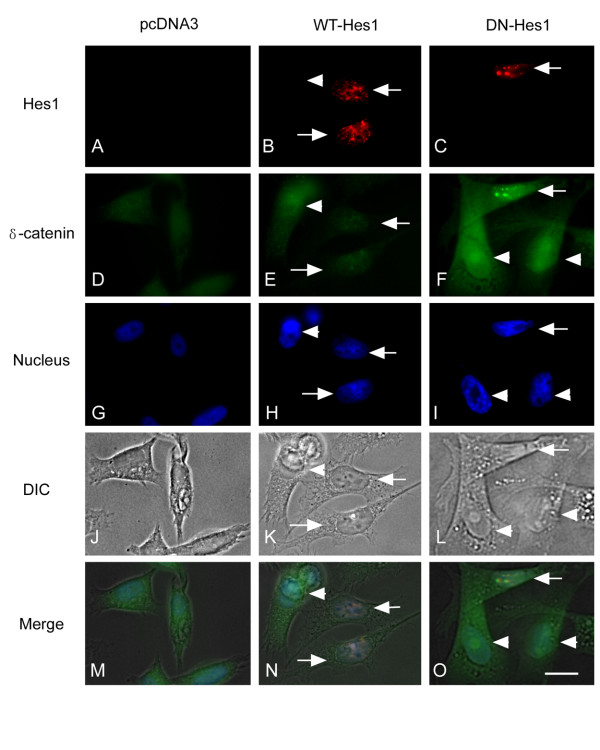
**Immunofluorescence light microscopy of PC3 cells transfected with WT-Hes1, DN-Hes1 or pcDNA3, respectively**. Red shows Hes1 protein stained by anti-flag-M5 antibody. Green shows δ-catenin stained by R1152 antibody. Blue shows nuclei stained with Hoechst 33258. Arrows (B, E, H, K and N): cells transfected with *WT-Hes1*; Arrows (C, F, I, L and O): *DN-Hes1*. Arrowheads: cells transfected with *pcDNA3*. Bar: 10 μm.

We then applied immunofluorescence light microscopy to investigate the expression and distribution of Hes1 and δ-catenin in CWR22-Rv1 and PC3 cells. Anti-flag-M5 antibody for flag-tagged Hes1 did not stain control, untransfected cells as expected (Figure 5A) but labeled strongly the nuclei when Hes1 was overexpressed in CWR22-Rv1 cells (Figure 5B and N, arrows). CWR22-Rv1 cells were clustered so we applied marking lines to better reveal the cell-cell boundary (Figure 5K and 5L). Anti-δ-catenin stained cytoplasm and cell-cell contacts in untransfected cells (Figure 5D), but the transfected cells showed a decreased intensity of anti-δ-catenin immunoreactivity (Figure 5E and N, arrows) when compared with the nearby untransfected cells (Figure 5E, arrowheads). The same results were obtained in PC3 cells that the overexpression of Hes1 reduced the anti-δ-catenin immunoreactivity (Figure 6B, E and N, arrows and arrowheads). However, when dominant negative Hes1 (*pcDNA-flag-DN-Hes1*) was transfected in either CWR22-Rv1 cells or PC3 cells, its inhibitory effects on the expression of δ-catenin were very weak, comparable to that of control, untransfected cells (Figure 5 and 6, C, F and O, compare arrows and arrowheads). Combined with the RT-PCR and Western blot data (Figure 4), these studies suggest that overexpression of wild type Hes1 can reduce the expression of δ-catenin in prostate cancer cells.

### Suppression of endogenous Hes1 expression increases δ-catenin expression in prostate cancer cells

**Figure 7:**
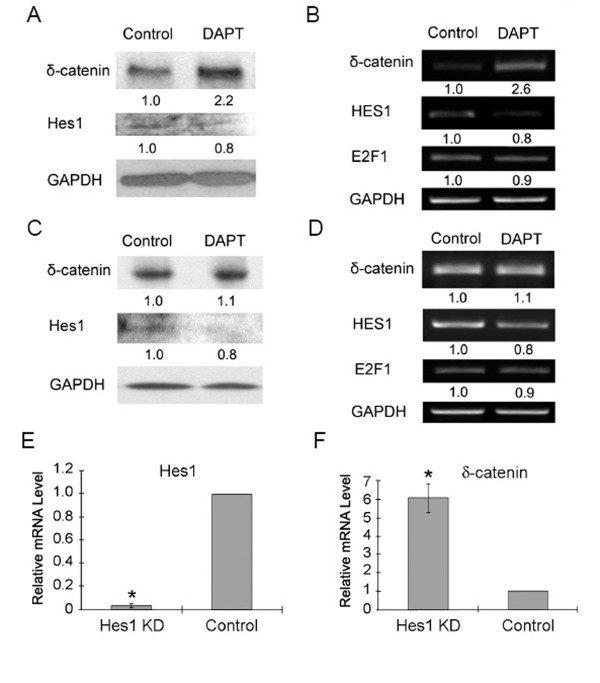
**Reduction of Hes1 corresponds to increased δ-catenin expression**. (A-D). Inhibition of γ-secretase decreases Hes1 expression while increasing δ-catenin expression. PC3 (A and B) and CWR22-Rv1 (C and D) cells were treated with (DAPT) or without (Control) the γ-secretase inhibitor DAPT at 20 μM for 48 hours. DAPT reduced Hes1 expression and increased δ-catenin expression. (A) Western blot analyses showing Hes1 and δ-catenin expression in PC3 cells with GAPDH as a loading control. (B) RT-PCR analyses for *Hes1, E2F1 *and *δ-catenin *in PC3 cells with *GAPDH *as PCR control. (C) Western blot analyses showing Hes1 and δ-catenin expression in CWR22-Rv1 cells with GAPDH as a loading control. (D) RT-PCR analyses for *Hes1, E2F1 *and *δ-catenin *in CWR22-Rv1 cells with *GAPDH *as PCR control. (E-F). siRNA against *Hes1 *suppressed Hes1 expression while increasing *δ-catenin *expression in PC3 cells. (E). siRNA against *Hes1 *nearly depleted *Hes1 *expression level compared to scrambled siRNA as a transfection control. (F). siRNA against *Hes1 *increased *δ-catenin *expression level by 6-fold compared to scrambled siRNA as a transfection control. KD: Knockdown. Two independent experiments were performed.

It was reported that γ-secretase inhibitor DAPT reduced Hes1 level in two prostate tumor cell lines, PC3 and LNCaP, in a dose-dependent manner [19]. After being treated with 20 μM DAPT for 48 h, Hes1 expression showed a decreasing trend at both the mRNA and protein level in PC3 cells (Figure 7A and 7B). Correspondingly, δ-catenin mRNA and protein levels in PC3 cells increased markedly (Figure 7A and 7B). The changes of *E2F1 *mRNA expression were less clear in PC3 cells (Figure 7B). These results were consistent with the notion that the relief of inhibitory function of Hes1 by DAPT may increase the δ-catenin expression in PC3 cells.

In CWR22-Rv1 cells, treatment with 20 μM DAPT for 48 h also reduced Hes1 expression but with little changes in *E2F1 *expression; the expression of δ-catenin in transcript and protein levels only showed a slight increase (Figure 7C and 7D, *p *< 0.05). To directly test the hypothesis that a reduction of Hes1 can lead to increases in δ-catenin expression, we applied siRNA against *Hes1 *to knock down endogenous *Hes1 *expression in PC3 cells (Figure 7E). Real-time PCR clearly showed that depletion of *Hes1 *mRNA increased *δ-catenin *transcript level by over 6-fold (Figure 7F).

### δ-Catenin shows a coordinated expression with E2F1 and Hes1 in NE-10 mouse model of prostate cancer

**Figure 8:**
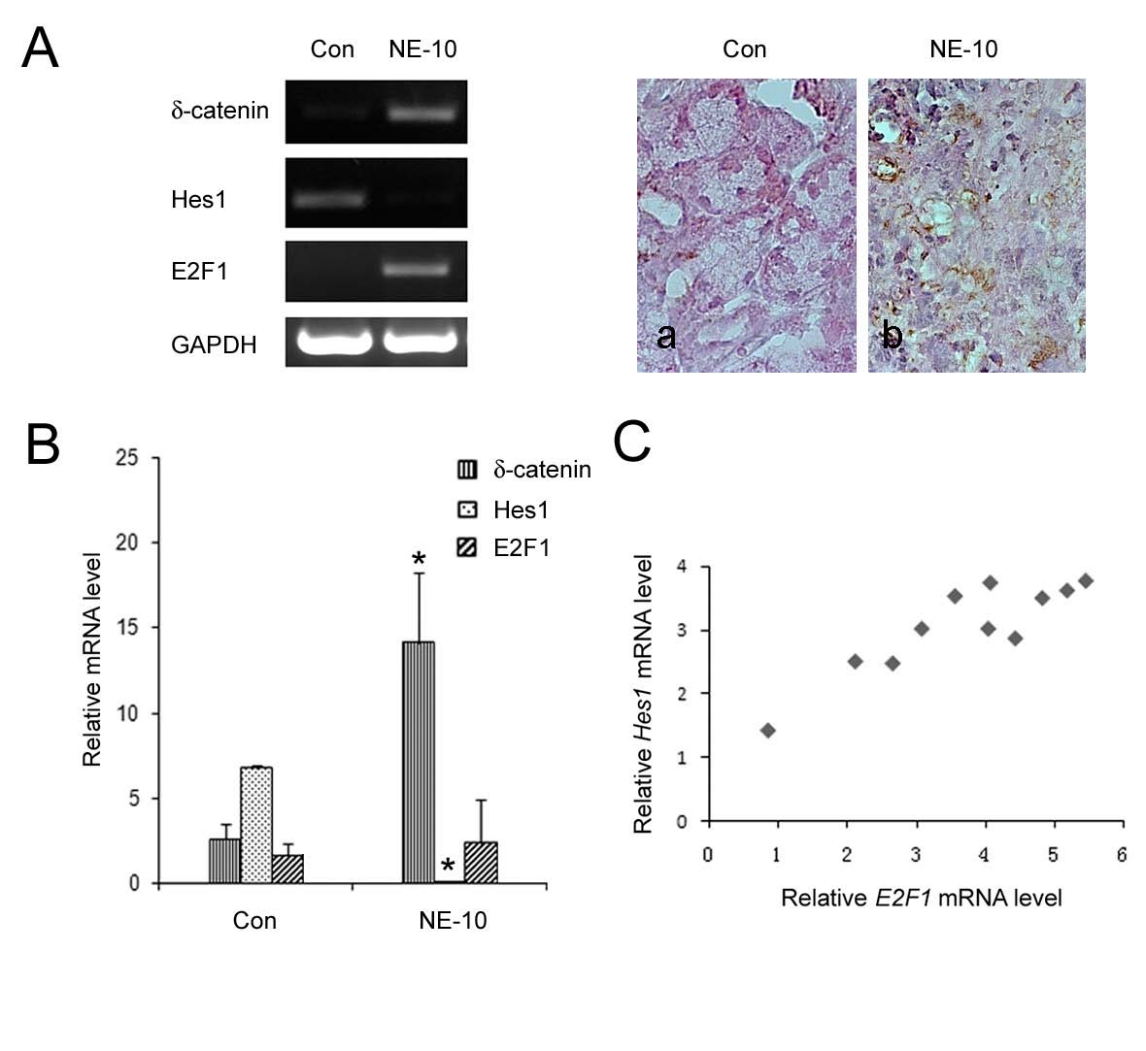
**Expression of transcriptional regulators of δ-catenin in NE-10 mice bearing prostate tumors**. (A) Left panel: RT-PCR of δ-catenin, Hes1 and E2F1 gene expressions in one representative set of normal prostate (10 week old CD1 mouse) and NE-10 tumor tissues [14]; Right panel: Immunohistochemistry showing increased δ-catenin expression in NE-10 mouse tumor specimen as compared to a normal mouse prostate. a. Normal mouse prostate tissue. b. NE-10 tumor tissue. Original magnification: x400. (B) Comparison of real-time qRT-PCR of *δ-catenin, Hes1*, and *E2F1 *gene expression between normal prostate and NE-10 tumors (*GADPH *as control). While *δ-catenin *transcript level was consistently increased, *Hes1 *expression was diminished. However, *E2F1 *transcript level varied greatly. (C) Comparison of real-time qRT-PCR of *Hes1 *and *E2F1 *gene expression among NE-10 tumors. Among the 11 NE-10 tumors, *E2F1 *transcription was relatively high when *Hes1 *level was correspondingly high. When the *E2F1 *transcription was relatively low, *Hes1 *expression was extremely low. These experiments were repeated twice, and the results were similar.

NE-10 is a tumor derivative allograft from the neuroendocrine prostate cancer mouse model 12T-10 [20]. Previous reports showed that compared to the normal mouse prostate, Hes1 expression was suppressed in NE-10 tumors [14]. This was confirmed in our studies (Figure 8A, left panel). Both RT-PCR and immunohistochemistry also showed that δ-catenin immunoreactivity was increased in NE-10 tumors when compared to that of normal prostate (Figure 8A, left and right panels). To determine whether a coordinated regulation of E2F1 with Hes1 on δ-catenin expression occurs *in vivo*, we have sought to compare the changes of δ-catenin expression along with *Hes1 *and *E2F1 *expression in NE-10 tumors and normal prostates. By using RT-PCR and real-time qRT-PCR, we found that *Hes1 *expression in NE-10 tumors was diminished whereas *δ-catenin *expression was correspondingly increased (Figure 8A and 8B). The majority of NE-10 tumors also showed an increased expression of *E2F1 *(Figure 8A, left panel). However, when *E2F1 *levels from all NE-10 tumors were examined collectively by real-time qRT-PCR, they were quite variable; it did not show statistically significant differences between the NE-10 tumors and normal prostate specimens (Figure 8B, n = 12). A closer examination led us to find that, among the 11 NE-10 tumors in which Hes1 expression was already very low, *E2F1 *transcription was relatively high when *Hes1 *level was correspondingly high (Figure 8C). When the *E2F1 *transcription was relatively low, *Hes1 *expression was extremely low (Figure 8C). These results were consistent with the notion that the changes of δ-catenin expression in NE-10 tumors could be the result of coordinated regulation of *Hes1 *and *E2F1 *expression.

### γ-Secretase inhibitors alter cell cycle progression and induce cellular processes in prostate cancer cells in culture

**Figure 9:**
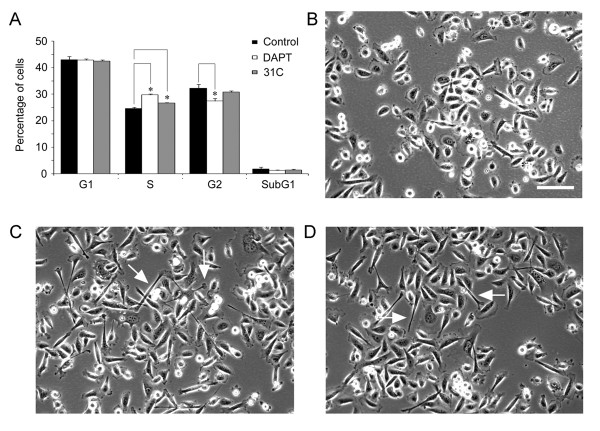
**Inhibition of γ-secretase altered PC3 cell cycle progression and increased cellular processes**. (A) Percentage of cell populations in each cell cycle stages (G1, S, G2/M and SubG1) of PC3 cells treated with or without γ-secretase inhibitors (DAPT or WPE-III-31C). * *p *< 0.05. (B-D) Morphology of PC3 cells treated for 48 hours with DMSO (B, control), DAPT (C) or WPE-III-31C (D). Note: Inhibition of γ-secretase showed increased number of cells with elongated shape or with extended cellular protrusions (Arrows) in PC3 cells (C and D). 31C: WPE-III-31C. Three independent experiments were performed. Bar: 50 μm.

Because γ-secretase inhibitor DAPT reduced Hes1 level in prostate cancer cell lines [19] (see also Figure 7), we sought to explore whether the γ-secretase inhibition-mediated increases in δ-catenin expression is accompanied by phenotypic changes with implications of neuroendocrine alterations. We examined the cell cycle profiles and the morphology of PC3 cells. After treatment with DAPT, there were no significant changes in G1 or SubG1 populations when compared to control PC3 cells (Figure 9A). However, there were significant increases (*p *< 0.05) in cell number in the S phase with corresponding decreases in the G2/M populations, indicating a delay in S/G2 transition (Figure 9A). Similar results were obtained in additional experiments using another γ-secretase inhibitor WPE-III-31C, although its effects were more moderate (Figure 9A).

PC3 cells in rapid growth phase showed typical epithelial cell morphology (Figure 9B). However, PC3 cells treated with DAPT or WPE-III-31C for 48 hours altered cell morphology. They displayed increased number of cells with elongated shape or with extended cellular protrusions (Figure 9C, DAPT; Figure 9D, WPE-III-31C; see arrows). These results are consistent with previously reported functions of Hes1 and δ-catenin and support a role of Hes1 suppression and δ-catenin expression in neuroendocrine tumor progression [21-23].

## Discussion

δ-Catenin expression is upregulated in most human prostatic adenocarcinomas [6] and other epithelial cancers [5]. Recently, Pax6 and E2F1 were identified as positive transcriptional regulators for δ-catenin in the central nervous system [24] and in prostate cancer cells [10]. However, whether there are pivotal negative transcriptional regulators for *δ-catenin *and how they may coordinate with positive regulators to control δ-catenin expression are unknown.

Transcriptional factor binding consensus analysis using Genomatix program http://www.genomatix.de/ revealed that *δ-catenin *promoter region contains potential binding sites of negative transcriptional regulators, RBP-Jkappa, Hes1, and p53 [10]. We have found that PC3 and CWR22-Rv1 are two prostate tumor cell lines with relatively high expression levels of both δ-catenin and Hes1.

While a single transcription activator or repressor less likely controls δ-catenin expression in prostate cancer cells, our current study supports the notion that Hes1 could negatively regulate the expression of δ-catenin in prostate cancer cells. Similar to the positive effects of E2F1 [10], Hes1 acts negatively on δ-catenin expression in a dose-dependent manner.

Hes1 expression is downregulated in non-metastatic cancer cell LNCaP compared with that in metastatic cancer cell C4-2B and may act as a tumor suppressor for primary prostate tumorigenesis [19,25]. In breast and pancreatic endocrine tumors, Hes1 was also downregulated [16,17]. However, in some tumors, Hes1 is upregulated during tumorigenesis, such as osteosarcomas [26,27]. As tumorigenesis is further underway, gene expression in cancer cells is deregulated and seems to be very complex and multi-faceted in nature. Hes1 may thus act as a tumor suppressor in one context and as an oncogene in another depending on the tumor types and the stages of cancer progression.

Notch-Hes1 axis in signaling is intricately modulated to control proliferation, differentiation, and apoptosis [28]. Hes1 is a target of Notch1 signaling, which is aberrantly activated in a variety of human cancers, including prostate, lung, colorectal, osteogenic, and breast carcinomas [12,13,26,29,30]. Hes1 may also contribute to osteoblast growth and differentiation by controlling transcription directly through interactions with transcriptional regulators [30]. The relationship between the aberrant expression of Hes1, E2F1, and δ-catenin in tumorigenesis is quite complex *in vivo *as well. In NE-10 neuroendocrine tumors [20] where δ-catenin expression was increased, Hes1 expression was diminished and E2F1 expression was increased. However, the extent of Hes1 reduction and E2F1 upregulation appeared to be tightly controlled in NE-10 tumors to avoid a complete shutdown of Hes1 expression with an overwhelming upregulation of E2F1 at the same time. One consequence of this coordination is for tumor cells to watch closely the expression level of δ-catenin during tumor progression. Therefore, it is possible that δ-catenin expression in NE-10 tumor is regulated both by its positive and negative transcriptional regulators.

These findings may have important functional implications for gene deregulation in tumor progression. It is well documented that Myc oncoprotein can activate E2F transcription factor, which can initiate both cell proliferation and apoptosis [31]. Earlier studies found that E2F1 transgenic mice showed increases in spontaneous tumor formation in the skin, but they also displayed an inhibition of tumor promotion by *O*-tetradecanoyl-phorbol-13-acetate (TPA) [32]. A closer examination of E2F1 expression using tissue microarray found that E2F1 was low in benign and localized prostate cancer, modestly elevated in metastatic lymph nodes from hormone-naive patients, and significantly elevated in metastatic tissues from hormone-resistant prostate cancer patients [33]. These studies suggest that in the early stages of prostate cancer development, E2F1 may be kept low to allow its oncogenic effects to be best accomplished with the cooperation of other oncogenic proteins, such as Myc, to outweigh its potential apoptotic effects. Quite interestingly, δ-catenin expression increased correspondingly from Gleason 4 to 6 and peaked at Gleason 8 prostatic adenocarcinomas, but reduced somewhat at Gleason 10 tumors, although its overall level was still higher than that of non-cancer prostatic tissues [6]. It is possible that in individual human prostate cancer cases, simultaneously higher δ-catenin and E2F1 expression level during early cancer development is not beneficial for tumor growth. On the other hand, an unusually high δ-catenin as well as E2F1 expression [33] could indicate the aggressiveness of tumor progression as they could signal that the tumors have already passed the very early stage of the oncogenic buildup.

## Conclusion

Our study shows that human homolog of *Drosophila *Hes1 negatively regulates δ-catenin (*CTNND2*) expression, alters cell cycle progression, and influences neuroendocrine-like cell morphology. These studies support coordinated regulation of δ-catenin expression by both the activating transcription factor E2F1 and repressive transcription factor Hes1 in prostate cancer progression.

## Methods

### Plasmids

The *Hes1 *expression plasmids (*pcDNA-flag-WT-Hes1*, *pcDNA-flag-DN-Hes1*), which contain rat *Hes1 *gene and the plasmid for *E2F1*, were described previously [10,18]. The construction of human *δ-catenin*-luciferase reporter vectors (BK1 and BK5), based on pGL3-Basic reporter vector (Promega), was previously reported [10]. pSV-β-Galactosidase control vector (Promega) or pcDNA3 (Invitrogen) was used as a control vector.

In *δ-catenin-luciferase *reporter vector BK5 (containing 744 bp of *δ-catenin *promoter) [10], there are four E-boxes (position in -425 to -437, -432 to -442, -493 to -506, and -631 to -643), which are potential binding sites for Hes1 (Figure 1A). These four E-boxes in *δ-catenin *promoter were mutated via site-directed gene mutagenesis one by one. The E-box in position -631 to -643 was mutated from ACGcgcgCGGCGA to ACGatatCGGCGA using mutation primer set (5'- CAGGAGAAGTGGAACGatatCGGCGAAGCGCCGCT-3' and AGCGGCGCTTCGCCGatatCGTTCCACTTCTCCTG). The obtained PCR product using wild BK5 vector as template was digested with DpnI, cloned to DH5α bacteria, and sequenced. The sequencing confirmed mutant BK5-M1 was used as template to mutate the second E-box at position -494 to -506, from CTGCcgcgCGCCG to CTGCatatCGCCG, using primer set (5'-GGAGGCTGAGGCTGCatatCGCCGCGGGAGGAG-3' and 5'- CTCCTCCCGCGGCGatatGCAGCCTCAGCCTCC-3'). The resulted mutant (BK5-M1, 2) vector mutated in the above two E-boxes was then used as template to mutate the third and fourth E-boxes at positions -425 to -437 and -432 to -442 using primer set (5'-AGGTGGCGCGGGCCGCatatGGGGCGCAGCTCGGGA-3' and 5'- TCCCGAGCTGCGCCCCatatGCGGCCCGCGCCACCT-3') from GGGCCGCcgcgGGGGCGC to GGGCCGCatatGGGGCGC. The final BK5-M1, 2, 3 mutant (BK5Mu) contains a 744 bp *δ-catenin *promoter mutated in all known 4 E-boxes and was used to evaluate the binding ability of Hes1 in *δ-catenin *promoter.

### Antibodies

The antibodies used were as follows: mouse monoclonal anti-δ-catenin Delta-30 (BD Bioscience), rabbit polyclonal anti-δ-catenin antibody R1152 raised against 435-530 amino acid and affinity purified essentially as described [3], rabbit anti-Hes1 polyclonal antibody (Millipore), mouse anti-flag antibody M5 (Kodak), mouse monoclonal anti-actin (Calbiochem), and mouse monoclonal anti-GAPDH mAb (6C5) (Calbiochem). Mouse monoclonal anti-δ-catenin/NPRAP/Neurojungin (J19) was a gift from Dr. Werner Franke.

### Cell culture, transfection and siRNA against Hes1

CWR22-Rv1 and PC3 human prostate cancer cell lines, as well as PZ-HPV-7 normal prostate cell lines were from ATCC and maintained in RPMI 1640 media supplemented with 10% FBS and 1% penicillin/streptomycin (CWR22-Rv1 and PC3), or in Keratinocyte-SFM media supplemented with EGF, bovine pituitary extract, and 1% penicillin/streptomycin (PZ-HPV-7) at 37°C in a 5% CO_2 _atmosphere. Cells were transfected using Lipofectamine Plus reagent (Invitrogen) or Fugene 6 reagent (Roche) according to the manufacturer's instructions. For Hes1 knockdown experiments, specific siRNAs directed against human *Hes1 *nucleotide sequences were obtained from Darmacon Technologies (USA). The ON-target plus smart siRNA oligonucleotide sequences were as follows: ACGAGAGCAAGAAUAAAU, AGGCUGGAGAGGCGGCUAA, UCAACACGACACCGGAUAA, and ACUGCAUGACCCAGAUCAA. A scramble siRNA was used as control.

### γ-Secretase inhibitor treatment and flow cytometry

Inhibition of endogenous Hes1 in PC3 and CWR22-Rv1 cell lines was achieved by a 48-hour treatment of 20 μM DAPT (N- [N-(3, 5-difluorophenylacetyl- L -alanyl)]-S-phenylglycine t-butyl ester) (Calbiochem), a peptidomimetic inhibitor of γ-secretase. Control plates were treated using the solvent DMSO (dimethylsulfoxide) (Sigma) with the same final concentration. For cell morphology and flow cytometry analyses, PC3 cells were incubated with 20 μM DAPT, 300 nM WPE-III-31C (a transition-state analog of γ-secretase) [34,35], or DMSO (control), respectively for 48 hours. Cells in multiple areas of culture plates were photographed for morphological analyses before they were collected with mild trypsinization and centrifugation. Cells were fixed in ice-cold ethanol and stained with 50 μg/ml propidium iodide containing RNase in the dark. The percentage of cells in each phase of the cell cycle (SubG1, G1, S, and G2/M) was determined by flow cytometry on a FACScan (BD Biosciences, Palo Alto, CA) with ModFit 3.1 software (Varity Software House, Topsham, ME). All data was presented as mean ± SEM and statistically evaluated with *t*-test. The confidence level was set at 95%.

### Luciferase reporter assay

Cell cultures in 12-well plates were transiently transfected using Fugene 6 reagent with expression vectors for several genes (*E2F1, WT-Hes1 *or *DN-Hes1*, luciferase gene, *GAL*) as indicated in the result section. Luciferase activity was measured 24 hours after transfection using the Luciferase Assay system (Promega) and normalized to β-galactosidase activity, which was used as control to determine transfection efficiency. All experiments were performed in quintuplicates. Whenever errors were displayed they represented standard deviation of mean (SDM) except in cell cycle measurements where SEM was employed.

### Electrophoretic mobility shift analysis (EMSA)

Nuclear extracts were prepared from PC3 or CWR22-Rv1 cells transfected with *pcDNA-flag-WT-Hes1 *using Fugene 6 reagent (Roche) 24 h before preparation according to instructions for NE-PER Nuclear and Cytoplasmic Extraction Reagents (Pierce Biotechnology, Rockford, IL). Oligonucleotides containing the HLH motif derived in *δ-catenin *promoter (5'-GGGCGAAGGCCCAGAGGCCTTCCTTGGCACATGTTTTGGGTTTCGTTTTTCAACAAGACT-3') [the E-box/HLH motif is underlined] (marked as ** in Figure 1A) and its reverse complement sequences were 3'-end labeled separately with Biotin 3' End DNA Labeling Kit (Pierce Biotechnology, Rockford, IL) and then annealed. Unlabeled shorter oligonucleotides (CCTTCCTTGGCACATGTTTTGGGTT) [the E-box/HLH motif is underlined] and its reverse complement sequences were annealed and used as competitors. Mutant probe was produced when E-box sequence CACATG was mutated to GTCTCA. EMSA reactions were performed according to instructions of LightShift Chemiluminescent EMSA Kit (Pierce Biotechnology, Rockford, IL). Nuclear protein extracts (2 μl) were incubated for 20 min at room temperature with 1 μl of non-specific competitor DNA Poly (dI·dC) and 20 fmol of biotin-labeled oligonucleotides. Competition assays were performed by mixing non-specific competitor DNA with 4 pmol unlabeled oligonucleotides (200-fold molar excess) and nuclear extracts before addition of probes. Hes-1 antibody (2 μl; Santa Cruz, CA) was used for disrupt-shift experiments. Normal goat IgG, Pax-6 antibody (2ul; Santa Cruz, CA), and δ-catenin antibody (2 μl; Transduction Laboratory, BD Biosciences) were used as a non-specific antibody. Antibodies were mixed with nuclear protein on ice for 20 min before the addition of probe DNA. Protein-DNA complexes were run on a 6% acrylamide gel, electrophoretically transferred to nylon membrane and visualized by Chemiluminescent Nucleic Acid Detection Module (Pierce Biotechnology, Rockford, IL).

### Chromatin immunoprecipitation (ChIP)

PC3 cells were collected and ChIP assay was performed to test Hes1 protein regulation on *δ-catenin *promoter using the Abcam ChIP kit (Abcam, Cambridge, MA) according to the manufacturer's instruction. Cross-linking was performed by adding formaldehyde to a final concentration of 1% at room temperature for 10 minutes and reaction stopped by the addition of 125 mM glycine. Cells were washed with ice-cold phosphate buffered saline containing 0.1 mM PMSF. Cell pellets, collected by centrifugation at 2000 rpm at 4°C, were resuspended in 1 ml of ChIP sonication buffer. DNA was sheared by sonication and the cell debris was pelleted by centrifugation at 14,000 g for 15 minutes. The pre-cleared whole cell extract was incubated with or without the antibodies, as described in the legend, at 4°C overnight. Immunoprecipitated, immune complexes were collected using anti-Hes 1 conjugated-Protein A. Anti-Histone 3 (ab1791) antibody and IgG were used as positive control and negative control, respectively. The primer sets of real-time PCR for *δ-catenin *promoter were as follows: Forward: 5'-CCTTCCAGCTTTCGCCTA G-3', Reverse: 5'-TCCACTTCTCCTGGTTTTCG-3'; GAPDH was used as control, the primers were as follows: Forward: 5'-AGAAACAGGAGGTCCCTACTCCC-3', Reverse: 5'-AGAGCGCGAAAGGAAAGA AAG CGT -3'.

### Mouse tissue preparation

The development of mouse neuroendocrine tumors (NE-10) was described before [14]. Male CD1 mice (Charles River Lab, Wilmington, MA) were obtained and used at 10 weeks of age. Mice were kept under pathogen free conditions and sacrificed by cervical dislocation according to the guidelines of East Carolina University Animal Use Protocol. The prostates were dissected into three different lobes (ventral, lateral-dorsal, and anterior lobe) under a dissecting microscope.

### Immunohistochemistry

For NE-10 and CD-1 mice prostate tissue immunohistochemistry, 5 μm tissue sections of formalin-fixed, paraffin-embedded blocks were deparaffinized and rehydrated; endogenous peroxidase was blocked by incubation with hydrogen peroxide. The sections were immunostained using rabbit anti-δ-catenin R1152 (1:100) (Abcam, Cambridge, MA) followed by streptavidin-biotin peroxidase method for detection. The immunostaining was done in Dako Autostainer (Carpinteria, CA) according to the manufacture instruction.

### RT-PCR and real-time qRT-PCR

Total RNAs were extracted from cultured cells or mouse tissues using an RNeasy mini kit (Qiagen) with residual genomic DNA removed by RNase-free DNase (Qiagen) treatment. 1~2 μg of total RNA was reverse-transcribed using Retroscript reverse transcription kit (Applied Biosystems).

For RT-PCR, the reaction was initiated using Advantage 2 polymerase or Advantage-GC 2 polymerase (Clontech). The following forward and reverse primers were used to produce gene specific fragments: human *δ-catenin*, forward 5'-ATGTTTGCGAGGAAGCCGC-3' and reverse 5'-GTCTGGTTGCTATGGTAGCTGGC-3'; mouse *δ-catenin*, forward 5'-GAGCTATGCCTGTCCCAGAC-3' and reverse 5'-AGCTGAGAAGGGGCTGTGT-3'; human *Hes1*, forward 5'-CAGCGAGTGCATGAACGAGGTGA-3' and reverse 5'- AGGTGCCGCTGTTGCTGGTGTAGA-3'; mouse *Hes1*, forward 5'-AAGAGGCGAAGGGCAAGAATAAAT-3' and reverse 5'-CCGGGGATGGGCACAAG-3'; rat *Hes1*, 5'-ACAGCCTCTGAGCACAGAAAGTCA-3' and reverse 5'-TGAGGAAAGCAAATTGGCCGTCAG-3'; human *Hey1*, forward 5'- TCGAGTTCGACTGGTTTCGCATCT-3' and reverse 5'- AGGTCTATAGGGCTTGCCAAGGTT-3'; human *E2F1*, forward 5'-ACTCCTCGCAGATCGTCATCATCT-3' and reverse 5'-GGACGTTGGTGATGTCATAGATGCG-3'; mouse E2F1, forward 5'-GCATCCAGCTCATTGCCAAGAAGT-3' and reverse 5'-TGGTGACAGTTGGTCCTCTTCCAT-3'; human *LEF-1*, forward 5'-CCCGCTTCCGCCCGCTGTCC-3' and reverse 5'-CGGGGTGTTCTCTGGCCTTGTCGT-3'; human *p53*, forward 5'-AGACCGGCGCACAGAGGAAGAGAA-3' and reverse 5'-CCCCGGGACAAAGCAAATGGAAGT-3'; human *CDK8*, forward 5'-AGAAGCTGCTTACCATGGACCCAA-3' and reverse 5'- TGGTGGAACTTGGCTACTGGACAT-3'; *human androgen receptor (AR)*, 5'-AGACGCTTCTACCAGCTCACCAA-3' and 5'- AGCTCTCTAAACTTCCCGTGGCAT-3'; human c-*Myc*, forward TCCACACATCAGCACAACTACGCA and reverse TCAGCCAAGGTTGTGAGGTTGCAT; human *GAPDH *(as control), forward 5'-GGGGAGCCAAAAGGGTCATCATCT-3' and reverse 5'- GACGCCTGCTTCACCACCTTCTTG-3'; mouse *GAPDH *(as control), forward 5'-AACTTTGGCATTGTGGAAGG-3' and reverse 5'- TGTGAGGGAGATGCTCAGTG-3'. PCR products were analyzed by electrophoresis in 1.0% agarose gel containing ethidium bromide and photographed under UV illumination.

For real time qPCR, reaction was initiated using IQ SYBR green supermix (Bio-Rad) in an iCycler iQ Multicolor Detection System (Bio-Rad). The relative mRNA levels of tested genes were calculated against control *GAPDH*. The following primers for real time qPCR were used: mouse *δ-catenin*, forward 5'- ACCTCGGGAAATGATCAGCCTCAA-3' and reverse 5'- TAGTTCCGTGGTAAGTGGCGTTGT-3'; mouse *Hes1*, forward 5'- CAACACGACACCGGACAAACCAAA-3' and reverse 5'- TGGAATGCCGGGAGCTATCTTTCT-3'; mouse *E2F1*, forward 5'-TCATGCCAGGAGACATCC-3' and reverse 5'-CAATACTGCTTCTTGCTCCA-3'; mouse *c-Myc*, forward 5'- TGCTGCATGAGGAGACA -3' and reverse 5'-TCGGGATGGAGATGAGC-3'; mouse *AR*, forward 5'- CTGCCTGATCTGTGGAGA-3' and reverse 5'- CAATGGTACAATCGTTTCTGC-3'; mouse *CDK8*, forward 5'- ACATTCTGGTACCGAGCT-3' and reverse 5'- CACCCTATAGCCCAAATATCAA-3'; and mouse *GAPDH *(as control), forward 5'-TCAACAGCAACTCCCACTCTTCCA-3' and reverse 5'- ACCCTGTTGCTGTAGCCGTATTCA-3'. The real-time PCR primer set for human genes were: human ***δ-**catenin*, forward 5'-GCCTCAGTCAAAGAACAGGA-3'and reverse 5'-AGCTTGCATCGCTCCA-3'; human *Hes1*, forward 5'-CTGAAGAAAGATAGCTCGCG-3' and reverse 5'-ACTTCCCCAGCACACTT-3'; human *E2F1*, forward 5'- CTCCGAGGACACTGACA-3' and reverse 5'- CACCATAACCATCTGCTCTG-3'; human *GAPDH*, forward 5'-ACAGTCAGCCGCATCTT-3' and 5'-GCCCAATACGACCAAATCC-3'.

### Immunofluorescence light microscopy

Cells on coverslips were fixed in 4% paraformaldehyde/phosphate buffered saline, permeabilized with 0.2% Triton X-100, blocked with 10% BSA in PBS, double immunostained with mouse anti-flag antibody M5 (1:600)/secondary antibody mouse Cy3 (1:400) and then rabbit polyclonal anti-δ-catenin antibody R1152 (1:50)/secondary antibody rabbit FITC (1:100). The nuclei were counter stained with Hoechst 33258 (2 μg/ml). The slides were mounted using Molecular Probe's Antifade medium and were analyzed under Zeiss Axiovert inverted fluorescent light microscope (Carl Zeiss) equipped with MetaMorph software (Molecular Devices).

### Western blot

Cultured cells were lysed in 10 mM HEPES, pH 7.3, 150 mM NaCl, 2 mM EDTA, 1% Triton X-100, 0.5% deoxycholate, 0.2% SDS with protease inhibitor cocktails. Equal amounts of protein samples were analyzed directly by SDS-PAGE. After proteins were transferred to nitrocellulose membranes (PGC Scientifics), Western blots were performed with appropriate antibodies and developed with ECL Western blotting detection reagents (Amersham).

## Competing interests

The authors declare that they have no competing interests.

## Authors' contributions

JL and JZ performed most of the experiments and contributed to the data analyses. KK provided δ-catenin promoter constructs. YHC provided real-time qRT-PCR analyses. JN performed immunohistochemistry experiments. TC and RM provided NE-10 samples and contributed to the design of the experiments. MW provided γ-secretase inhibitor and provided suggestions for the experiments. QL contributed to the design of the entire study and the editing of the manuscript. All authors read an approved the final draft.
